# Exposures to Conditioned Flavours with Different Hedonic Values Induce Contrasted Behavioural and Brain Responses in Pigs

**DOI:** 10.1371/journal.pone.0037968

**Published:** 2012-05-25

**Authors:** Caroline Clouard, Mélanie Jouhanneau, Marie-Christine Meunier-Salaün, Charles-Henri Malbert, David Val-Laillet

**Affiliations:** 1 INRA, UR1341 ADNC (Alimentation & Adaptations Digestives, Nerveuses et Comportementales), Saint Gilles, France; 2 INRA, UMR1348 PEGASE (Physiologie, Environnement et Génétique pour l’Animal et les Systèmes d’Élevage), Saint Gilles, France; 3 Agrocampus Ouest, UMR1348 PEGASE (Physiologie, Environnement et Génétique pour l’Animal et les Systèmes d’Élevage), Rennes, France; Barnard College, Columbia University, United States of America

## Abstract

This study investigated the behavioural and brain responses towards conditioned flavours with different hedonic values in juvenile pigs. Twelve 30-kg pigs were given four three-day conditioning sessions: they received three different flavoured meals paired with intraduodenal (i.d.) infusions of 15% glucose (F_Glu_), lithium chloride (F_LiCl_), or saline (control treatment, F_NaCl_). One and five weeks later, the animals were subjected to three two-choice feeding tests without reinforcement to check the acquisition of a conditioned flavour preference or aversion. In between, the anaesthetised pigs were subjected to three ^18^FDG PET brain imaging coupled with an olfactogustatory stimulation with the conditioned flavours. During conditioning, the pigs spent more time lying inactive, and investigated their environment less after the F_LiCl_ than the F_NaCl_ or F_Glu_ meals. During the two-choice tests performed one and five weeks later, the F_NaCl_ and F_Glu_ foods were significantly preferred over the F_LICl_ food even in the absence of i.d. infusions. Surprisingly, the F_NaCl_ food was also preferred over the F_Glu_ food during the first test only, suggesting that, while LiCl i.d. infusions led to a strong flavour aversion, glucose infusions failed to induce flavour preference. As for brain imaging results, exposure to aversive or less preferred flavours triggered global deactivation of the prefrontal cortex, specific activation of the posterior cingulate cortex, as well as asymmetric brain responses in the basal nuclei and the temporal gyrus. In conclusion, postingestive visceral stimuli can modulate the flavour/food hedonism and further feeding choices. Exposure to flavours with different hedonic values induced metabolism differences in neural circuits known to be involved in humans in the characterization of food palatability, feeding motivation, reward expectation, and more generally in the regulation of food intake.

## Introduction

Flavours are perceived during food consumption [1] and result from the combination of a taste and odour, through the stimulation of the gustatory system as well as the orthonasal and retronasal olfactory systems respectively [2], [3]. All animal species have the ability to associate the flavour of a specific food with the consequences of its ingestion and can modulate further food intake via the establishment of conditioned food preferences [4], [5] or aversions [4], [6]. Conditioned food/flavour preference and aversion have been widely studied, especially in rats. Usually, preference conditioning is experimentally induced by pairing an unknown flavour with abundant caloric supply, e.g. gastric glucose infusions [7]–[11], while aversion conditioning is induced by pairing an unfamiliar flavour with a visceral infusion of emetic substances, e.g. lithium chloride [12]–[16].

In rats, numerous brain lesion studies investigated the brain structures involved in the acquisition of flavour preference and aversion, such as the amygdala (AMY) [10], [17]–[19] and the insular cortex (IC) [20]–[23]. Unfortunately, those studies often focused on the only first steps of preference and aversion learning processes, i.e., detection of the conditioned stimulus (CS), detection of the visceral unconditioned stimulus (US), association between the US and the CS (e.g., role of the parabrachial nucleus [24], [25]). Athough Touzani et al. [10], [11] reported that the AMY and the lateral hypothalamus were not involved in the expression of conditioned flavour preferences in rats, less is known about the brain structures that are involved in the last steps of those processes, i.e., retrieval of the learning when the CS is further encountered and expression of the appropriate behaviour. Consequently, there is a need for studies to investigate the brain structures that are involved in the recall of conditioned learning during subsequent exposure to the conditioned flavour (CS).

Thanks to the emergence of functional brain imaging techniques, several brain structures involved in the processing of hedonic information during olfactogustatory stimulations have been characterized in rats, primates and humans. Both the IC [26] and the AMY [27] are known to be involved in the evaluation of food stimuli hedonism, but with contradictory data. Some papers reported that the IC is activated during pleasant odour exposure [28], [29] and the AMY during aversive stimuli [30]–[32], while others noticed some activation of the two structures during both unpleasant and aversive food/taste stimuli exposure [33]–[36]. The basal nuclei also play an important role during the processing of food hedonism. For instance, the nucleus accumbens (NAcc) is activated by pleasant food stimuli [37], while cerebral responses in the caudate (CAU) and the putamen (PUT) decrease with decreasing reward value of stimuli in humans [38]. Additionally, the dorsolateral prefrontal cortex (DLPFC) [39], the orbitofrontal cortex (OFC) [28], [36], [40]–[42], the cingulate cortex [26], [28], [31], [37], [38], [42] and the parahippocampal cortex (PHC) [38] are involved in the processing of the hedonic value of taste and/or odour stimuli. Lastly, the temporal gyrus participates in the recognition of food-related vs. food-unrelated stimuli and is involved in the perception of pleasant taste [39], [43].

Gaultier et al. [29] performed the very first study aimed at exploring the pigs’ brain metabolism during exposure to flavours with contrasted hedonic values acquired after aversive or positive flavour conditioning using Single Photon Emission Computed Tomography (SPECT). Compared to a control condition (no flavour), the perception of a preferred or an aversive flavour triggered brain responses in the prefrontal cortex, the temporal gyrus, some limbic structures, the IC, as well as the AMY and several basal nuclei. These findings suggest that similar structures are involved in the recognition of food-related flavours with different hedonic values in pigs and humans. However, two major limitations can be pointed out in the study of Gaultier et al. [29]. First, the only positive reinforcement during conditioning was the positive postingestive consequences (food hedonism and caloric supply) provided by the CS (i.e., the meal), but there was no additional positive oral or visceral reinforcement. Second, during the imaging sessions, the control stimulation was provided by exposure to unflavoured air and saliva. As the control stimulation was not a food-related stimulus, it is difficult to determine whether the differences of brain metabolism recorded after the perception of the conditioned flavours compared to the control stimulation were triggered by the perception of flavours with contrasted hedonic values, or by the perception of a food-related flavour compared to a non-food condition.

To continue and complete this work, three main objectives were defined in the present study: 1) to modulate food intake of pigs by pairing a flavoured meal with intraduodenal (i.d.) infusions with different putative hedonic values, 2) to check the acquisition of the conditioning and its persistence a month after learning by studying animals’ food preference during repeated two-choice feeding tests with the flavoured meals, and 3) to investigate, via brain Positron Emission Tomography (PET), the brain activity patterns in some predefined structures during subsequent exposure to flavours with contrasted hedonic values in anaesthetized animals. We hypothesized that: 1) the animals would exhibit contrasted behavioural patterns after different visceral reinforcement during conditioning, 2) food preferences would be shaped by conditioning on a long-term basis, and 3) subsequent exposure to the conditioned flavours would induce contrasted activity patterns in some brain structures involved in the processing of hedonic judgment and discrimination during sensory stimulations, and especially during gustatory and/or olfactory stimulations.

## Materials and Methods

### Ethics Statement

The experiments presented in this paper were conducted in accordance with the current ethical standards of the European Community (Directive 86/609/EEC), Agreement No. A35–622 and Authorizations No. 01894 and No. 35–88. The Regional Ethics Committee in Animal Experiment of Brittany has validated the entire procedure described in this paper (b–2009–DVL–01).

### Animals and Housing

A total of twelve 30-kg Large White × Piétrain female pigs were used in this study. The pigs were individually housed in pens (150×60×80 cm) and had free access to water. A chain was suspended in each pen to enrich the environment of the animals and fulfil their natural disposition to play. The room was maintained at approximately 24°C with a 13∶11-h light-dark cycle. The animals were fed daily at 09∶00 with 1 kg of pelleted meal (3.63 kcal/g) composed of 30% barley, 30% wheat, 25% hulled oat, 6% bran, 5% molasses, 1.5% bi-calcic phosphate, 1.5% calcium carbonate, 0.5% salt and 0.5% vitamin complement. In order to accustom the animals to the experimental oiled meal, the pelleted meal was supplemented with 10 mL of vegetable oil (Phodé Laboratories, Terssac, France) per kg of food, the vehicle enabling the adjunction of essential oils in the food during conditioning (see *Experimental procedure* section).

### Surgery

After a 24-h fasting period, the pigs were preanaesthetised with an intramuscular injection of ketamine (15–20 mg/kg, Mérial, Lyon, France), then put on isoflurane (3–5% v/v, Isoflurane Belamont, Nicholas Piramal, London, UK) anaesthesia and subjected to a tracheal intubation. A surgical level of anaesthesia was maintained by isoflurane (2–3% v/v) delivered by a mechanical ventilator and analgesia was obtained by intravenous injection of a morphinic agent (Fentanyl 4 mL, 1.4 mL/min, Renaudin, Paris, France). Heart rate was continuously monitored throughout surgery using a pulse oxymeter (Ohmeda oxymeter, GE Healthcare Clinical Systems, Limonest, France). Normocapnia was controlled by an infrared capnometer (Amstrong capnometer, Gambo Engström, Bromma, Sweden). A midline laparotomy was performed under aseptic conditions. A catheter was inserted into the proximal duodenum, tunnelled under the skin and exteriorized between the shoulders for further i.d. infusions during food conditioning. The animals were allowed one week to recover from surgery before the beginning of the experiments. During the recovery week, the animals were exclusively fed with the oiled meal and were accustomed to eat their meal in 30 min.

### Conditioned and Unconditioned Stimuli Preparation

The conditioned stimuli were flavoured meals. The three flavoured meals were elaborated by the adjunction in pelleted meal of essential oils of thyme (T; 0.4%), orange (O; 0.15%), or cinnamon (C; 0.1%) diluted in vegetable oil (Phodé Laboratories, Terssac, France), with 10 mL of additive per kg of meal. At these dilutions, the animals normally consume as much thyme-, orange- and cinnamon-flavoured meal [29]. The unconditioned stimuli were produced by an i.d. injection of glucose (Glu), lithium chloride (LiCl) or saline (NaCl) 5 min before the end of a 30-min meal. The putative positive reinforcement was induced by an i.d. injection of 150 mL of glucose 15% (90 kcal; Glu treatment). The negative reinforcement was induced by an i.d. injection of 50 mL of LiCl 8%, followed by 100 mL of saline – NaCl 0.9% (LiCl treatment). As NaCl has no particular postingestive effect, the control treatment was induced by an i.d. injection of 150 mL of saline – NaCl 0.9% (NaCl treatment). Solutions were injected with a peristaltic pump connected to the duodenal catheter, and the injection rate was 10 mL/min.

### Experimental Procedure

The study was carried out in three successive batches, each of them lasting seven weeks and composed of a conditioning period, a testing period and a brain imaging period. Four animals were studied in each batch, in which the presentation order of the flavours was counterbalanced to avoid any bias.

#### Conditioning sessions

After the recovery week, the animals were subjected to four three-day conditioning sessions with the flavoured meals. For each conditioning session, on day 1, the animals were fed during 30 min the cinnamon- (batch 1), thyme- (batch 2) or orange-flavoured meal (batch 3), on day 2, the animals were fed the thyme- (batch 1), orange- (batch 2) or cinnamon-flavoured meal (batch 3) and on day 3, the animals were fed the orange- (batch 1), cinnamon- (batch 2) or thyme-flavoured meal (batch 3). Each day, a third of the pigs received the LiCl treatment, a third of the animals received the Glu treatment, and the last third received the NaCl treatment. Consequently, at the end of the conditioning period, the animals have been subjected to a total of four repetitions of each kind of conditioning, that is a treatment (LiCl, Glu, NaCl) paired with a specific flavoured meal (T, C, O; e.g. C_LiCl_/T_NaCl_/O_Glu_ or C_Glu_/T_LiCl_/O_NaCl_ or C_NaCl_/T_Glu_/O_LiCl_). Each day of conditioning, the meal was removed after 30 min and refusals were weighed.

#### Two-choice feeding test sessions

After two weeks of conditioning, the pigs were subjected to three two-choice feeding tests to assess their preferences for the different flavoured meals (F_LiCl_, F_Glu_ and F_NaCl_). On day 1, 2 and 3, the animals could choose between the thyme- and the cinnamon-flavoured meals, the thyme- and the orange-flavoured meals, and the orange- and the cinnamon-flavoured meals, respectively. The two different meals were presented in a two-part trough containing 1 kg each. They were presented at 09∶00 to the animals, and during 30 min. Then, the two-part trough was removed and refusals were weighed. No i.d. injection was given during these preference tests. Meal distribution in the troughs was interchanged over days and animals to avoid any bias. The same three two-choice feeding tests were repeated one month after the end of the conditioning to ensure that the conditioned learning did not extinguish before the end of the brain imaging sessions. Meal distribution in the trough was interchanged compared to the first testing session.

### Behavioural Analyses

During the conditioning sessions, behavioural observations were carried out during the 30 min following the end of the meal. Behaviours were recorded using the scan-sampling method (1 observation every 30 sec) and the Pocket Observer® software (Noldus, Wageningen, Nederland) installed in a pocket PC (iPAQ 214, Hewlett-Packard, Palo Alto CA, USA). The behavioural repertoire was adapted from the study of Gaultier et al. [29]: bars-focused activity (bites or licks the pen’s bars), ground-focused activity (licks, paws, rubs the ground), self-directed activity (scratches or licks its own body), chain-focused activity (chews or plays with the chain), trough-focused activity (bites or licks the trough although there is no food in it), drinks, vomits, urinates/defecates, chews (with no food in the mouth), no activity and other activities. Additionally, four postures were recorded: standing, sitting, kneeling down and lying. Behavioural observations were also carried out during the 30-min two-choice test meals. The same method and the same items were used, with one additional item “eats” (chews with the head at the trough when there is still food in it). The trough used by the animal when eating was systematically specified in order to determine the time spent in each trough during the meal.

### Brain Imaging Procedure

After the first session of two-choice food tests, the animals (three out of four per batch) underwent three brain imaging sessions to investigate the brain metabolism during flavour exposure (F_LiCl_, F_Glu_ and F_NaCl_). The brain imaging modality used to investigate the cerebral glucose metabolism (CGM) was the PET of ^18^F-fluorodesoxyglucose (^18^FDG, CIS bio international, France).

#### Animal preparation and olfactogustatory stimulation

After a 24-h fasting period, the animals were anaesthetized and subjected to a tracheal intubation following the same procedure as that described above (see *Surgery* section). The animals were placed in a Head First Prone position on the bed of a whole body, high-resolution PET and a venous catheter was inserted in their left ear in order to inject the radiolabel. The ears and eyes of the animals were sealed with cotton and surgical tape respectively, in order to minimize auditory and visual stimulations. Animals’ body temperature was maintained at least at 37°C by using a heating blanket.

**Figure 1:**
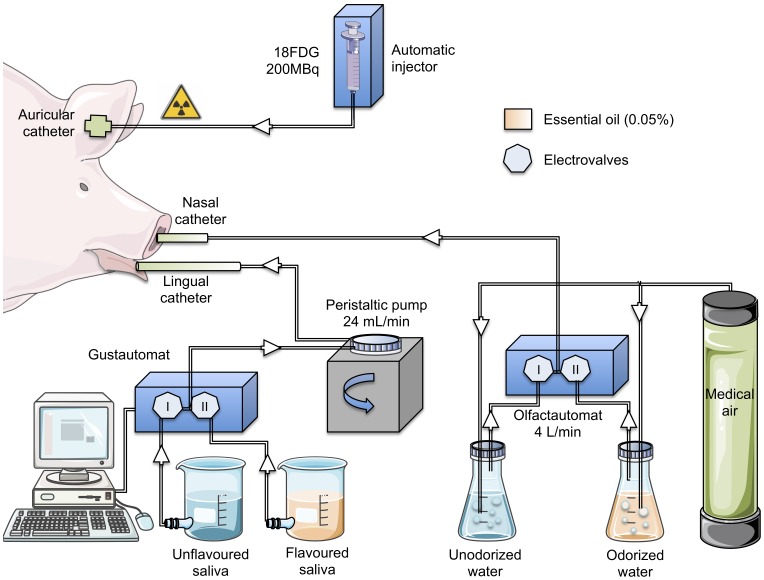
Experimental device and paradigm designed to perform olfactogustatory stimulations for brain imaging in anaesthetised pigs. The illustrations used to make this figure were obtained from the “Servier Medical Art” website, http://www.servier.fr/servier-medical-art.

The olfactogustatory stimulation was performed with computer-assisted automats designed in our laboratory (Figure 1). The olfactory stimulation consisted in diffusing a nonodorized or an odorized air (0.05% essential oil) into the pig’s right nostril (4 L/min). As the animals were intubated and mechanically ventilated, the diffused air could not come out from the mouth. Consequently, the olfactory stimulation was performed via one of the two nostrils to let the air flow through the nasal cavity. The choice to perform the stimulation via the right nostril rather than the left nostril, however, has been done arbitrarily. A tube was inserted in the right nostril of the animal and connected to a device composed of a medical air cylinder connected to a flow meter and a two-way circuit of bottles equipped with a system of electronic valves. One of the bottles contained unodorized tap water and the other contained odorized water (0.05% essential oil). The gustatory stimulation consisted in irrigating the pig’s tongue (24 mL/min) with an unflavoured or a flavoured artificial saliva (0.05% essential oil; for the saliva composition, see [44]). A tube was positioned on the middle of the tongue and connected to a computer-operated automat developed in our laboratory (Gustautomat, INRA, St Gilles, France, see [29]) and inspired by the Taste–o–Matic designed by Hellekant’s group [45]. The animals were subjected to a neutral olfactogustatory stimulation (i.e., nonodorized air and unflavoured saliva) for 5 min to accommodate the mucosa thermoreceptors and mechanoreceptors to the stimulation. Then, the diffusion of odorized air and flavoured saliva was performed for 15 min. The stimulation was ended by a 15-min neutral stimulation.

#### Data acquisition

The radiolabel (^18^FDG, 200MBq) was injected 5 min after the beginning of the olfactogustatory stimulation procedure. PET data were acquired on a CTI/Siemens HR+ Scanner in 3D mode (Siemens ECAT, 962, HR+). A 30-min 3-dimensional (3D) emission scan was performed 45 min after the radiolabel injection using an axial FOV of 15.52 cm. It was corrected by a 15-min transmission scan using rotating ^68^Ge rods. Following scatter, dead time and random corrections, PET transaxial images were obtained by iterative reconstruction using a ramp filter (Kernel FWHM  = 6 mm) providing 63 contiguous slices. Spatial resolution after reconstruction was 0.64 mm per pixel in the x and y directions and 2.42 mm per pixel in the z axis. Pixel depth encoding was performed using the Standard Uptake Value (SUV) method.

#### Image processing

The data were analyzed with statistical parametric mapping (SPM8, Wellcome Trust Centre for Neuroimaging, London, UK) implemented in MATLAB 7.1 (The Mathworks Inc., Natick MA, USA). The pre-processing of the PET images obtained in this study was realized in 6 steps. The images were first manually reoriented (pitch  = −1.57077, roll  = 3.14159). The spatial coordinates were then centered compared to a reference point (x_0_, y_0_, z_0_, posterior commissure). The images were masked to remove the extracerebral matter, and the coordinates were realigned on a PET template. The images were then spatially normalized and the normalization was restricted to 12-parameter affine transformations in order to minimize deformations of the original images. A second narrower masking was then performed to eliminate more finely the extracerebral matter. Finally, spatially normalized images were smoothed using a Gaussian filter set at 4×4×4 mm full width at half maximum. Eleven male and female pigs of approximately 35 kg different from those used in this experiment were used to build the PET template. PET images were acquired and processed as described above.

### Statistical Analysis

#### Statistical behaviour analysis

Data were analyzed with the Statview software 4.57 (Abacus Concepts Inc., USA). When Kolmogorov-Smirnov tests showed that the data presented a Gaussian distribution, parametric tests were performed. The conditioning consumption data were thus analysed using two-way repeated measures ANOVA followed by simple main effects tests when appropriate. The consumption data obtained during the two-choice tests were analysed using paired *t*-tests. The behavioural activity data were analysed using non parametric Wilcoxon tests. When multiple comparisons were performed, a Bonferroni correction was applied. Otherwise, the significant level for all analyses was set at *P*<0.05.

#### Statistical image analysis

The regional ^18^FDG uptake was standardized to the mean global uptake using proportional scaling in order to minimize interindividual differences in global CGM. The F_NaCl_, F_Glu_ and F_LiCl_ PET images were compared together using paired *t-*tests. The three contrasts (F_Glu_ – F_NaCl_, F_LiCl_ – F_NaCl_ and F_LiCl_ – F_NaCl_) presented hereafter show the bidirectional differences of brain metabolism, that is both higher and lesser CGM responses of one treatment compared to another. For practical reason and in each contrast, we systematically decided to compare the brain metabolism triggered by the perception of the less preferred flavour to that triggered by the perception of the more preferred flavour relatively to the behavioural responses during two-choice tests. Variances were considered unequal. The dependency and heteroscedasticy induce different error covariance components that were estimated using REML (Restricted Maximum Likelihood) and used to adjust the statistics and degrees of freedom during inference. By default, SPM uses weighted least squares to produce Gauss-Markov or Maximum likelihood estimators using the non-sphericity structure specified at this stage (SPM8 User Manual). The error variances were 1.075 for F_NaCl_ images, 0.966 for F_Glu_ images and 0.955 for the F_LiCl_ images, and the error covariances were 0.423 for the F_NaCl_/F_Glu_, 0.37 for the F_NaCl_/F_LiCl_, and 0.282 for the F_Glu_/F_LiCl_. A Small Volume Correction (SVC) analysis was performed with SPM8 on the regions of interests (ROIs) selected upon the *a priori* hypotheses presented in the introduction. With this analysis, that allows for voxel to voxel comparisons within restricted ROIs, we managed to identify the voxels for which the activity was statistically different between treatments in the ROIs. An uncorrected value of *P* = 0.05 was set as the threshold (extent threshold of 5 voxels).

Regression analyses were also performed to investigate a possible relationship between the brain metabolism in the ROIs obtained for the F_Glu_, F_NaCl_ and F_LiCl_ stimulations and the food consumption data. Two sets of consumption data were used for these regression analyses: 1) the amount of the F_Glu_, F_NaCl_ and F_LiCl_ food consumed during the last session of conditioning, and 2) the total amount of the F_Glu_, F_NaCl_ and F_LiCl_ food consumed during the two-choice tests performed one week after conditioning. These data were used to calculate a regression with the images obtained during the F_Glu_, F_NaCl_ and F_LiCl_ stimulations (each image was associated with the amount of food consumed during the conditioning or the two-choice tests). An uncorrected value of *P* = 0.05 was set as the threshold (extent threshold of 5 voxels).

The statistical analysis with SPM8 produced a listing of voxels for which the activation (CGM) differed between treatments. Each voxel was associated with a set of coordinates (x y z) corresponding to its spatial location in the CA-CP (*commissura anterior*-*commissura posterior*) plane with CP set as the origin. The ROIs chosen for the SVC analysis were anatomically identified on the basis of a 3D digitized pig brain atlas developed in our laboratory [46], and selected upon the *a priori* hypotheses presented in the introduction. Consequently, the ROIs included the structures (bilaterally) that are known to be involved in the evaluation of sensory stimuli valence, that is some prefrontal and frontal structures (the OFC, the DLPFC and the anterior prefrontal cortex (APFC)), the cingulate cortex, the PHC, the IC, the temporal gyrus, the AMY, and the basal nuclei (the CAU, the globus pallidus (GP), the NAcc and the PUT).

## Results

One out of the 12 animals was excluded from the study because it showed a generalized aversion for food, regardless of the flavour or the treatment associated with the meal, after only one pairing between the meal and the LiCl injection. A total of 11 and 9 animals were used for behavioural and brain imaging analyses, respectively.

### Behavioural Results

Before conditioning, there was no difference in the average amount of each flavoured food consumed (O: 834±85 g, T: 781±75 g, C: 874±45 g, *F*(2,10) = 0.92, *P* = 0.42).

#### Consumption and behaviour during conditioning sessions

**Figure 2:**
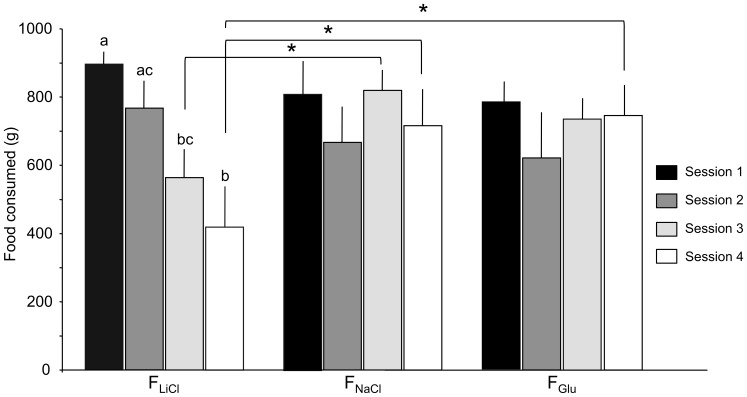
Quantity of food (g) consumed during the four conditioning sessions. During the conditioning period, the animals were given a 30-min flavoured meal associated with NaCl, LiCl or Glucose (Glu) duodenal injection. Data are presented with means and standard errors. Significant simple mean effects are indicated with asterisks and letters. An asterisk indicates a significant difference between two treatments during a single conditioning session (* *P*<0.05). Two different letters indicate a significant difference between two conditioning sessions for the same treatment (*P*<0.01).

The food consumption data are presented in Figure 2. The two-way within subjects ANOVA showed no global effect of the treatment (*F*(2,20) = 1.82, *P* = 0.19), but a significant global effect of the conditioning session (*F*(3,30) = 6.32, *P*<0.01) in that the pigs consumed more food during the first session than during the fourth (*P*<0.05) session; other comparisons were not significantly different. There was also a significant session-treatment interaction (*F*(6,60) = 9.48, *P*<0.001). Simple mean effect tests revealed that the F_LiCl_ food intake decreased over sessions in that the pigs consumed less of the F_LiCl_ food during the third (*P*<0.01) and fourth (*P*<0.001) sessions than during the first session, and during the fourth session than during the second session (*P*<0.001). The pigs also consumed less of the F_LiCl_ food than of the F_NaCl_ food (*P*<0.05) during the third and fourth sessions, and than the F_Glu_ food (*P*<0.05) during the fourth session only.

**Figure 3:**
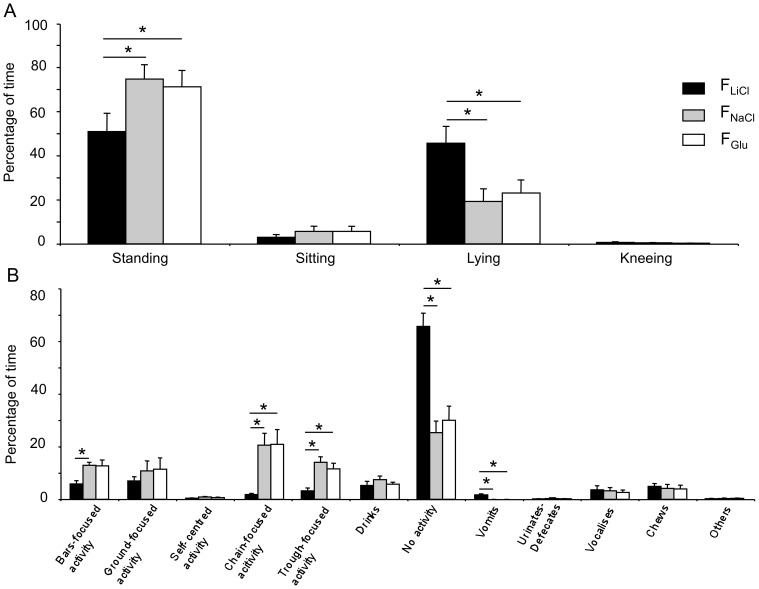
Behavioural observations performed during the conditioning sessions. Body postures (A) and behavioural activity (B) recorded during 30 min after a meal associated with NaCl, LiCl or Glucose (Glu) duodenal injection. Data are presented with means and standard errors. Significant differences between two treatments (*P*<0.05) are indicated with an asterisk.

There was no difference in the general activity exhibited by the animals after they received the NaCl or the Glu treatments (*P*>0.05). After the LiCl reinforcement, the animals spent less time standing (NaCl: *z* = 2.76, *P*<0.016; Glu: *z* = 2.5, *P*<0.016) and more time lying (NaCl: *z* = 2.76, *P*<0.016; Glu: *z* = 2.67, *P*<0.016) than after the NaCl or the Glu reinforcements (Figure 3a). They also spent more time inactive (NaCl: *z* = 2.93, *P*<0.016; Glu: *z* = 2.85, *P*<0.016) and less time in exploratory and playing activities (bars-focused, chain-focused or trough-focused activities) than after the NaCl or the Glu treatments (Figure 3b). The animals also spent 2% of their time vomiting whereas this behaviour was not expressed after the NaCl or the Glu treatments. A total of 2.1±0.4 vomiting occurrences were observed during the 30 min following the LiCl injection, with the first occurrence being observed 11.5±1.2 min after the beginning of the injection.

#### Consumption and behaviour during the two-choice feeding tests

**Figure 4:**
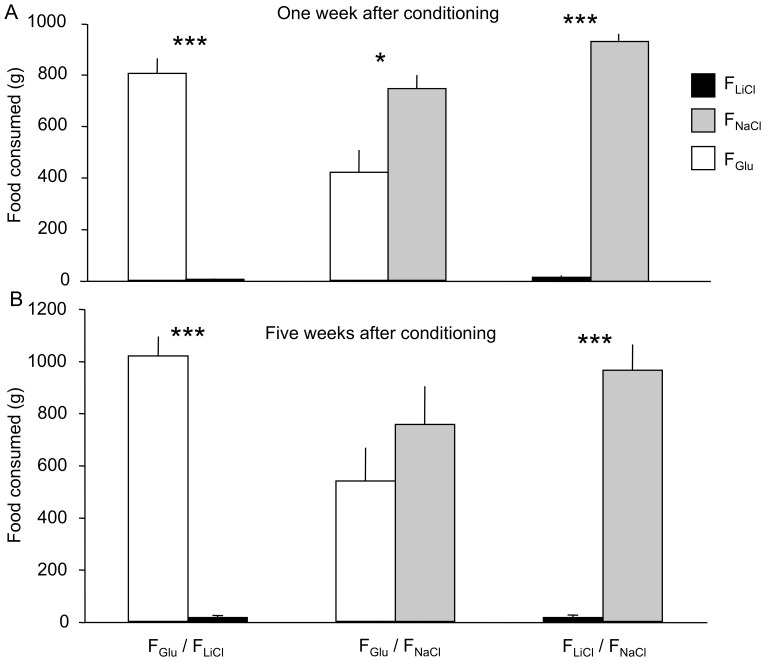
Quantity of flavoured food (g) consumed during the 30-min two-choice tests. The tests were carried out one week (A) and five weeks (B) after conditioning. Data are presented with means and standard errors. The following symbols are used * *P<*0.05; ** *P<*0.01.

During the two-choice feeding tests performed one week after conditioning (Figure 4a), the animals consumed significantly more of the F_NaCl_ (*t*(10) = 32.52, *P*<0.001) or F_Glu_ food (*t*(10) = 14.16, *P*<0.001) than of the F_LiCl_ food. The animals also consumed more of the F_NaCl_ food than of the F_Glu_ food (*t*(10) = 2.65, *P*<0.05). The animals spent significantly less time with the head in the trough containing the F_LiCl_ food than in the trough containing the F_NaCl_ (F_LiCl_: 2 ± 1%, F_NaCl_: 92 ± 3%, *z* = 2.93, *P*<0.01) or the F_Glu_ food (F_LiCl_: 1 ± 1%, F_Glu_: 86 ± 5%, *z* = 2.93, *P*<0.01). The animals also had a tendency to spend more time with the head in the trough containing the F_NaCl_ food than in the trough containing the F_Glu_ food (F_NaCl_: 61 ± 6%, F_Glu_: 38 ± 6%, *z* = 1.96, *P*<0.1). During the two-choice feeding tests performed one month after the conditioning (Figure 4b), the animals consumed significantly more of the F_NaCl_ (*t*(10) = 9.56, *P<*0.001) or F_Glu_ food (*t*(10) = 13.36, *P<*0.001) than of the F_LiCl_ food, but they did not consumed more of the F_NaCl_ food than of the F_Glu_ food anymore (*t*(10) = 0.85, *P = *0.42).

### Brain imaging results

The results of the SVC analysis in some brain regions for which differences of CGM were found for the three types of contrast are summarized in Tables 1 and 2.

**Table 1:** Regions that were more activated in the F_LiCl_ condition than in the F_NaCl_ and F_Glu_ conditions, and in the F_Glu_ condition than in the F_NaCl_ condition.

		F_LiCl_ – F_NaCl_	F_LiCl_ – F_Glu_	F_Glu_ – F_NaCl_
Middle temporal gyrus	L	2.47 (−18 −12 10)	1.94 (−18 −12 10)	
Inferior temporal gyrus	L	2.12 (−18 3 3)		2.31 (−22 5 9)
Inferior temporal gyrus	R		2.11 (18 3 3)	
Superior temporal gyrus	L	4.02 (−16 −8 12)	2.58 (−18 −9 11)	3.87 (−18 −3 15)
Parahippocampal cortex	L	2.00 (−16 −3 −2)		
Parahippocampal cortex	R		2.35 (14 −6 6)	
Dorsal posterior cingulate cortex	R	2.44 (4 −4 21)	2.42 (4 −5 19)	
Ventral posterior cingulate cortex	L		1.74 (−2 −5 14)	
Insular cortex	L			2.63 (−12 28 9)
Insular cortex	R		2.53 (22 7 13)	
Nucleus accumbens	R	1.78 (4 19 −4)	1.93 (2 18 −0)	
Caudate nucleus	R		2.20 (8 11 8)	
Globus pallidus	R	2.14 (8 17 −1)	2.62 (10 11 5)	
Putamen	L			1.86 (−6 27 −2)
Putamen	R	2.00 (8 19 −1)	2.36 (12 9 7)	
Amygdala	L	1.98 (−16 4 3)		
Amygdala	R		2.74 (16 4 2)	

The threshold for significance was set at *P<*0.05 (uncorrected). The *t*-value of the peak of maximal intensity is indicated for each cluster. The stereotaxic coordinates (x y z, in mm) of the peak in the CA-CP (*commissura anterior-commissura posterior*) plane with CP set as the origin are indicated in parentheses. L, left; R, right.

**Table 2:** Regions that were less activated in the F_LiCl_ condition than in the F_NaCl_ and F_Glu_ conditions, and in the F_Glu_ condition than in the F_NaCl_ condition.

		F_LiCl_ – F_NaCl_	F_LiCl_ – F_Glu_	F_Glu_ – F_NaCl_
Dorsolateral prefrontal cortex	L			1.79 (−4 31 9)
Anterior prefrontal cortex	L	2.40 (−6 30 2)	2.81 (−6 30 1)	1.85 (−0 29 −0)
Anterior prefrontal cortex	R	1.98 (4 32 2)	1.94 (4 35 2)	1.85 (0 29 −0)
Orbitofrontal cortex	L	1.92 (−0 23 3)		
Orbitofrontal cortex	R	1.87 (0 23 3)		1.82 (0 21 3)
Inferior temporal gyrus	L		1.80 (−22 5 9)	
Inferior temporal gyrus	R			2.57 (18 3 3)
Superior temporal gyrus	R	1.86 (22 −8 16)		
Parahippocampal cortex	L	2.72 (−8 −10 4)		1.96 (−10 −10 5)
Parahippocampal cortex	R			2.61 (14 −6 5)
Dorsal posterior cingulate cortex	L			3.42 (−4 1 17)
Dorsal posterior cingulate cortex	R	3.28 (2 4 17)		3.09 (0 3 18)
Dorsal anterior cingulate cortex	L			2.03 (−2 29 8)
Ventral anterior cingulate cortex	L	2.56 (−2 2 15)		2.42 (−2 1 15)
Ventral anterior cingulate cortex	R	2.62 (2 3 15)		
Insular cortex	L	2.30 (−8 31 3)	2.40 (−8 30 3**)**	
Insular cortex	R	1.83 (14 27 10)	2.10 (14 27 10)	2.10 (22 9 13)
Caudate nucleus	R			2.18 (8 11 8)
Globus pallidus	L	2.42 (−12 12 3)	2.00 (−6 15 1)	
Globus pallidus	R			2.05 (10 12 5)
Putamen	L	2.31 (−12 12 4)	2.48 (−6 27 −2)	
Putamen	R			2.11 (16 7 5)
Amygdala	L	2.05 (−12 11 −0)		2.10 (−10 11 −1)
Amygdala	R			3.07 (16 4 3)

The threshold for significance was set at *P<*0.05 (uncorrected). The *t*-value of the peak of maximal intensity is indicated for each cluster. The stereotaxic coordinates (x y z, in mm) of the peak in the CA-CP (*commissura anterior-commissura posterior*) plane with CP set as the origin are indicated in parentheses. L, left; R, right.

#### F_LiCl_ compared to F_NaCl_ or F_Glu_

**Figure 5:**
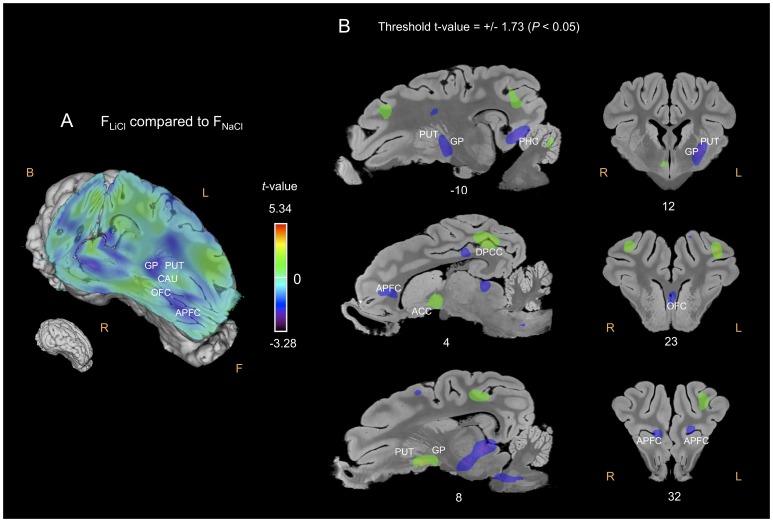
Cerebral glucose metabolism (CGM) differences obtained for the F_LiCl_ flavour compared to the F_NaCl_ flavour. (A) Three-dimensional skinned representation of the pig’s brain with global CGM differences found in the F_LiCl_ *vs* F_NaCl_ contrast. The (x y z) coordinates are indicated below the representation. (B) Sagittal and coronal MRI sections showing significant CGM differences in the F_LiCl_ *vs* F_NaCl_ contrast. The threshold for significance was set at *P<*0.05 (uncorrected). The x or y coordinates are indicated below each section. Positive *t-*values (green, yellow and red) indicate more activation in the F_LiCl_ condition than in the F_NaCl_ condition, while negative *t-*values (blue and purple) indicate more deactivation in the F_LiCl_ condition than in the F_NaCl_ condition. F, Front; B, Back; R, Right; L, Left; APFC, anterior prefrontal cortex; OFC, orbitofrontal cortex; CAU, caudate nucleus; GP, globus pallidus; PUT, putamen; DPCC, dorsal posterior cingulate cortex.

**Figure 6:**
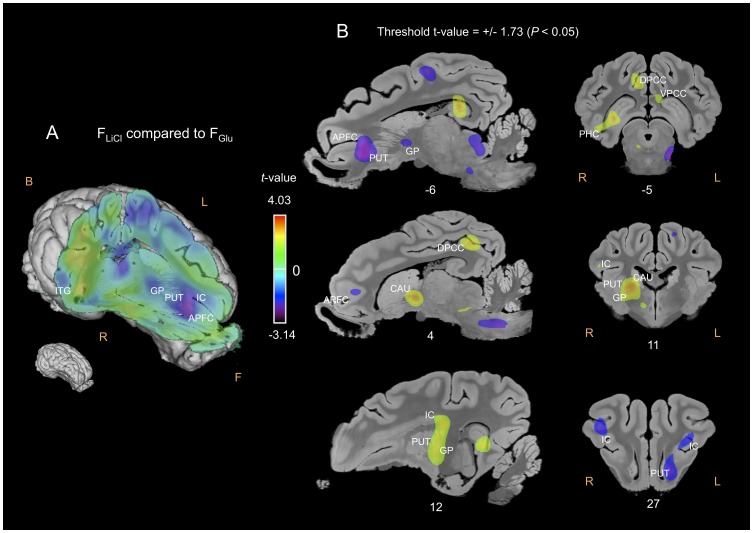
Cerebral glucose metabolism (CGM) differences obtained for the F_LiCl_ flavour compared to the F_Glu_ flavour. (A) Three-dimensional skinned representation of the pig’s brain with global CGM differences found in the F_LiCl_ *vs* F_Glu_ contrast. The (x y z) coordinates are indicated below the representation. (B) Sagittal and coronal MRI sections showing significant CGM differences in the F_LiCl_ *vs* F_Glu_ contrast. The threshold for significance was set at *P<*0.05 (uncorrected). The x or y coordinates are indicated below each section. Positive *t-*values (green, yellow and red) indicate more activation in the F_LiCl_ condition than in the F_Glu_ condition, while negative *t-*values (blue and purple) indicate more deactivation in the F_LiCl_ condition than in the F_Glu_ condition. F, Front; B, Back; R, Right; L, Left; IC, insular cortex; ITG, inferior temporal cortex. Other abbreviations: see Figure 5.

The APFC was significantly less activated in the F_LiCl_ condition than in the F_NaCl_ (Figure 5) or F_Glu_ (Figure 6) conditions, and the OFC in the F_LiCl_ condition than in the F_NaCl_ condition. Conversely, the PHC, the posterior cingulate cortex (PCC) and the AMY were more activated, while the anterior cingulate cortex (ACC) and the CI were globally less activated in the F_LiCl_ condition than in the F_NaCl_ or F_Glu_ conditions. As for the basal nuclei, the right NAcc, GP and PUT were more activated, whereas the left PUT and GP were less activated in the F_LiCl_ condition than in the F_NaCl_ or F_Glu_ conditions. The right CAU was also more activated in the F_LiCl_ – F_Glu_ contrast (Figure 6). Compared to F_NaCl_ and F_Glu_, the perception of F_LiCl_ induced higher CGM responses in the left (superior, middle and inferior) temporal gyrus.

#### F_Glu_ compared to F_NaCl_

**Figure 7:**
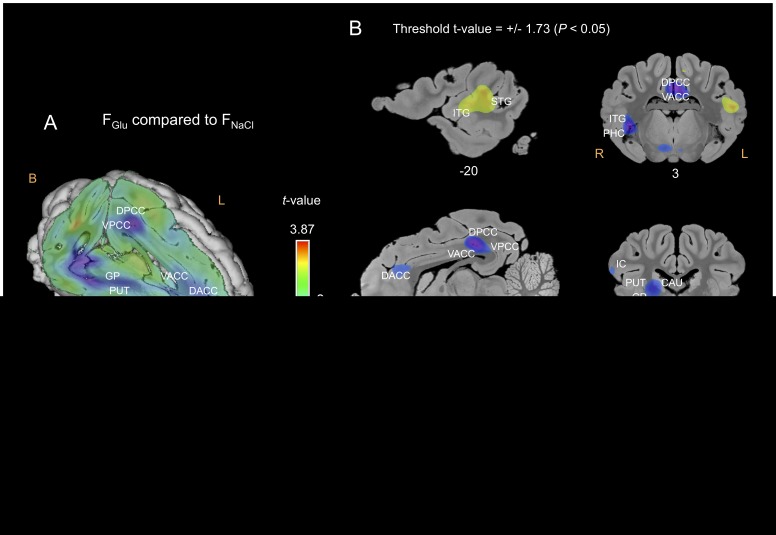
Cerebral glucose metabolism (CGM) differences obtained for the F_Glu_ flavour compared to the F_NaCl_ flavour. (A) Three-dimensional skinned representation of the pig’s brain with global CGM differences found in the F_Glu_ *vs* F_NaCl_ contrast. The (x y z) coordinates are indicated below the representation. (B) Sagittal and coronal MRI sections showing significant CGM differences in the F_Glu_ *vs* F_NaCl_ contrast. The threshold for significance was set at *P<*0.05 (uncorrected). The x or y coordinates are indicated below each section. Positive *t-*values (green, yellow and red) indicate more activation in the F_Glu_ condition than in the F_NaCl_ condition, while negative *t-*values (blue and purple) indicate more deactivation in the F_Glu_ condition than in the F_NaCl_ condition. F, Front; B, Back; R, Right; L, Left; OFC, orbitofrontal cortex; AMY, amygdala; STG, superior temporal gyrus; PHC, parahippocampal cortex; DACC, dorsal anterior cingulate cortex; VACC, ventral anterior cingulate cortex; VPCC, ventral posterior cingulate cortex. Other abbreviations: see Figures 5 and 6.

The APFC, the right OFC, the left DLPFC, the PHC and some parts of the cingulate cortex were less activated in the F_Glu_ – F_NaCl_ contrast (Figure 7). The right CAU, GP and PUT were less activated, while the left PUT was more activated in the F_Glu_ condition than in the F_NaCl_ condition (Figure 7). The left inferior and superior temporal gyrus was more activated and the right temporal gyrus was less activated in the F_Glu_ condition than in the F_NaCl_ condition (Figure 7).

#### Regression analyses between behavioural and brain imaging data

**Figure 8:**
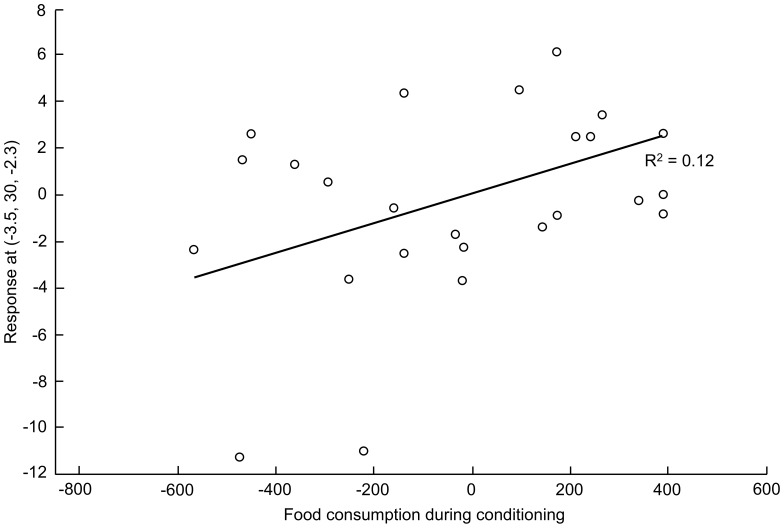
Relationship between the quantity of food consumed during the last conditioning session and brain metabolism for the voxel with the highest *t*-value (2.52) in the left anterior prefrontal cortex. Least-square regression line: R^2^ = 0.11709. The (x, y, z) coordinates of the voxel are indicated in the y-axis legend. The statistical value for the voxel is *P* = 0.009. The open circles indicate the adjusted data (% error) for the subjects.

The brain metabolism in 18 and 11 structures out of 34 was correlated with the quantity of food consumed during the last session of conditioning and food consumption during the preference tests performed 1 week after conditioning, respectively. Hereafter, we focused on the ROIs for which regression analysis was significant at *P*<0.01 for at least one voxel – the stereotactic coordinates [x y z] of the voxel with the highest *t*-value are indicated. Five out of the 6 voxels for which the metabolism was correlated with consumption data were located in the left hemisphere. The amount of food consumed during conditioning was significantly correlated with metabolism in the left ([−4 30 −2], *t = *2.5, *P* = 0.009; Figure 8) and right ([2 34 −2], *t = *2.6, *P* = 0.005) APFC, the left DLPFC ([−4 41 9], *t = *2.6, *P* = 0.008) and the left CAU ([−6 9 9], *t = *2.7, *P* = 0.007). The amount of food consumed during preference tests was significantly correlated with metabolism in the left APFC ([−6 30 3], *t = *2.8, *P* = 0.005) and the left IC ([−8 30 3], *t = *2.7, *P* = 0.007).

## Discussion

### Flavour Preference and Aversion Conditioning

Behavioural data showed that after the LiCl conditioning, the animals spent more time lying and inactive and less time expressing exploratory, rooting and playing activities than after the Glu and NaCl (control) reinforcements. A reduction of activity and an increase of the time spent lying are known to be indicative of discomfort and to reflect the expression of a malaise in various species [47]–[49]. Similarly, as playing behaviour has been suggested to be a positive indicator of welfare in juvenile individuals [50], a decrease of the chain-focused activity in our study is likely to indicate a decrease of well-being. According to these behavioural indicators, we assume that the LiCl treatment induced a state of ill-being in the pigs, which resulted in the development of a robust and persistent aversion for the associated flavoured meal. This aversion was confirmed by the systematic avoidance of the F_LiCl_ food during the subsequent two-choice feeding tests, a result that confirms previous data indicating that LiCl infusions induced strong food aversions in pigs [29]. On the other hand, no difference in the behavioural activities and food consumption was reported between the Glu and the NaCl treatments during conditioning, which might suggest that the glucose infusion was not more reinforcing than the saline infusion. Besides, preference tests indicated that the F_NaCl_ food was significantly preferred over the F_Glu_ food, at least one week after conditioning. Though unexpected, the lower preference found for the F_Glu_ in the present study suggests that a visceral glucose infusion might be perceived as a relatively negative reinforcement by pigs.

Different hypotheses might explain our inability to condition a glucose-induced preference. First, we injected a fixed dose of glucose, regardless of the quantity of food consumed. Although some studies also induced strong preferences for flavoured solutions paired with fixed doses of glucose (6 mL: [8]; 10 mL: [51]), several studies rather used a glucose amount directly proportional to the quantity of solution consumed, with a fixed ratio of 1∶1 [7], [10], [11], [52], [53]. Therefore, this suggests that the infusion of a dose of glucose adapted to the quantity of food consumed would have come to better results. Second, the amount of glucose injected might have been insufficient to induce a preference only based on energy supply. In the present study, the energy supply provided by 15% glucose infusions represented approximately 3 kcal/kg, while, in average, the amount of 8 or 16% glucose injected in rodents represented approximately 7 to 8 kcal/kg (e.g., [8], [52]). Consequently, the amount of energy injected was approximately 2.5 times lesser than in previous studies in rodents. Moreover, the conditioned stimulus was a caloric flavoured meal, not a non-caloric flavoured beverage. The postingestive reinforcing effect of glucose might have been in competition with or just overlapped by the stronger postingestive reinforcing effects of food and may explain our inability to develop a preference for the F_Glu_ food compared to the F_NaCl_ food, which was reinforcing in itself. Further trials with a greater amount of glucose injected would likely result in a successful preference conditioning.

### Neurobiological Determinants

In the second part of the study, we investigated, in predetermined ROIs, the differences of brain metabolism triggered by exposure to the conditioned flavours, i.e., the aversive flavour (F_LiCl_), the less preferred flavour (F_Glu_) or the preferred flavour (F_NaCl_). Three main findings emerged from our study: 1) exposure to aversive and less preferred flavours triggered lesser activation in the prefrontal lobe and 2) a lateralized pattern of activity in the basal nuclei and, in a lesser extent, in the temporal gyrus and, 3) exposure to the aversive but not to the less preferred flavour triggered higher activation in the PCC and the left AMY.

#### Negative flavour perception triggered lesser activation in the prefrontal cortex

The APFC was bilaterally less activated during exposure to both the aversive and less preferred flavours. Some authors reported that the APFC [29], as well as the OFC [30], [31], [33], was activated by both aversive and pleasant flavour perception in pigs and humans, suggesting that the prefrontal cortex might be involved in the recognition of food-related flavours rather than in the characterization of flavour palatability. The OFC, however, is known to be involved in the passive perception of odours but also in the active smelling of odours (hedonic and familiarity judgments; [28], [54]) and is implicated in the processing of reward [55]. As activation in the OFC is correlated with pleasantness ratings of the stimuli in humans [42], a lesser activation during exposure to a less preferred and/or aversive flavour was expected. In humans, Rolls et al. [28] also demonstrated that pleasant odours induced more activation in the medial OFC than unpleasant odours, while Gaultier et al. [29] reported that the deactivation in the OFC was larger during aversive than during preferred flavour perception in pigs.

Previous studies in humans reported activation in the left DLPFC during perception of a pleasant taste [39], [56], which is consistent with its lesser activation during the perception of a less preferred flavour (F_Glu_) in our study. The prefrontal cortex, and especially the left DLPFC, is involved in the treatment of feeding signals and is known to modulate food intake by sending inhibitory inputs to the orexigenic network to suppress hunger [57]–[59]. These results suggest that the perception of a flavour with a relatively low hedonic value is likely to modulate the inhibitory inputs sent to the orexigenic system, as well as further food intake. As the perception of the aversive flavour did not trigger similar brain responses, further investigation is needed to understand to what extent the level of aversiveness of the stimuli is determinant in the modulation of the DLPFC activity.

#### Negative flavours triggered lateralized patterns of activity in specific brain structures

We demonstrated that the perception of the aversive flavour induced lesser activation in the left basal nuclei compared to the control and less preferred conditions, while the perception of the less preferred flavour triggered lesser activation in the right basal nuclei compared to the control condition. As the basal nuclei are an integrant part of the reward system and mediate numerous goal-directed behaviours, including emotions, motivation, and cognition [55], lesser CGM during exposure to negative stimuli was quite expected. Besides, Small et al. [38], [60] reported that activation in the CAU and the PUT was correlated with pleasantness ratings of the stimuli and/or the motivation to eat (e.g., chocolate). Surprisingly, in our study, the perception of the aversive flavour also triggered higher activation in the right NAcc, GP, CAU and PUT, while the perception of the less preferred flavour triggered higher activation in the left PUT.

Numerous studies reported asymmetric brain activity during exposure to pleasant or unpleasant stimuli, although the results are not consistent. Henkin and Levy [61] reported that the smell of odours considered as unpleasant generally triggered greater activity in the right than in the left hemisphere, which is concordant with higher activation of the right basal nuclei found in our study during the perception of an aversive flavour. Gaultier et al. [29] found that the perception of a preferred flavour compared to an aversive flavour triggered activation in the left CAU, PUT, and GP in pigs, which is consistent with our finding that an aversive stimulation triggered lesser activation in left PUT or GP. Moreover, in our study, the correlation found between food consumption and brain metabolism, especially in left brain structures including the APFC, DLPFC, CAU and left IC, supports general knowledge admitting that the left hemisphere is involved in emotional processing of odours and hedonic judgments, while the right hemisphere is rather involved in the processing of odour familiarity and recognition [62]. Although we found that the perception of an aversive and/or a less preferred flavour mostly induced higher CGM responses in the left temporal gyrus, some studies in humans showed that activation during food or pleasant taste stimulation is higher in the left cortical regions, such as the superior temporal cortex [36], [39] known to be involved in the perception of taste [39]. As exposed here, scientific data are quite contradictory as for the lateralization of brain responses to sensorial stimulations [58], [63] and further studies are needed to extricate the relationships between brain lateralization and the processing of stimuli with contrasted hedonic values.

#### The perception of an aversive flavour triggered specific brain activations

The perception of the highly aversive flavour induced higher CGM responses in the AMY, the PHC and the PCC, whereas the perception of the less preferred flavour did not. In humans, the AMY, which is involved in the hedonic processing of olfactory and gustatory stimuli [30], [64], has been found to be activated during exposure to aversive odorants [30] and tastes [31], and it appeared that the amplitude of activation in the left AMY is correlated with the level of perceived aversiveness in humans [40]. In their meta-analysis, Costafreda et al. [32] reported that the AMY is activated by aversive rather than positive stimuli. All sensory stimuli with strong emotional value, however, are likely to induce AMY activation, regardless to the valence of the stimuli (pleasant and aversive) [33], [64], [65], although the responses are often less consistent with positive stimuli than with aversive stimuli [35]. As for the PCC, Small et al. [38] reported that it was more activated when patients eating chocolate rated it as highly pleasant or highly aversive than when they rated it neutral. According to Maddock [66], they concluded that the PCC was rather activated by stimuli with a high (positive or negative) emotional valence than by stimuli with a low or neutral emotional valence. Consequently, our results seems to corroborate the finding of Small et al. [38] in humans.

It is worth noting that we also found that different part of the cingulate cortex (e.g. the ACC), as well as the PHC, were less activated during perception of both aversive and less preferred flavours. Those structures are involved in the processing of olfactory perception [67] and in the emotional evaluation of sensory stimuli [26], [38], and the activation of the ACC is correlated with the pleasantness ratings of odours [28], [37], [42]. In their review, Haber and Knuston [55] reported that the ACC is highly associated with reward and strongly connected to the basal nuclei and consequently considered as an integrant part of the reward circuit. Deactivation in the PHC and the ACC during perception of the aversive and less preferred flavours was thus expected, especially since Reiman et al. [68] found that the ACC was involved in the experience of unpleasant emotions, while the PHC was rather activated by pleasant taste [39].

Lastly, we noticed that the IC was predominantly less activated during the perception of aversive or less preferred flavours. The IC is a multimodal structure receiving projections from the olfactory system (in monkeys: [69]), and is considered as the primary taste cortex [33], [36], [70]. Some studies reported that the IC is activated in response to olfactory stimulations [67], and especially, but not exclusively, to pleasant odour perception [28], though, other studies mentioned that the IC is rather activated during unpleasant and aversive gustatory stimulations [31], [33]. All together, these findings suggest that the IC might be involved in the recognition of flavours rather than in the processing of the stimulus hedonism or, that distinct parts of the IC are differentially implicated in the processing of aversive or pleasant sensory stimuli.

### Conclusion

In conclusion, we demonstrated that postingestive visceral stimuli can modulate the flavour/food hedonism and further feeding choices. We performed here one of the first studies highlighting considerable similarities in the pig’s and human’s brain metabolism during the processing of the hedonic value of sensory stimuli. As expected, exposure to flavours with different hedonic values induced some metabolism differences in neural circuits that have been identified in humans to be involved in the characterization of food palatability, flavour identification and more generally, in the regulation of food intake. The present study also complemented a previous study published by our group [29], which was the very first to describe unconscious brain responses during flavour exposure in pigs. These results are promising in terms of biomedical research applied to human nutrition and show that the pig is a good model to study the behavioural and neurobiological determinants of food intake. However, our study has some limitations requiring further investigations. First, while LiCl i.d. infusions induced a strong long-lasting flavour aversion, 15% glucose infusions failed to condition a flavour preference. As sweet taste enhances the effect of caloric supply [71], adding glucose directly in the food may enable to enhance its reinforcement value and condition a clear flavour preference to study the specific cerebral responses triggered by the perception of a highly pleasant flavour in pigs. Second, in the present study, the small number of animals prevented us from finding brain metabolism differences when correction was made for multiple comparisons. A complementary study using an improved paradigm and an increased number of pigs should result in the establishment of a persistent conditioned flavour preference and in a substantial improvement of the statistical power for brain imaging analyses.
